# Supplementary material for: Mild Endurance Exercise during Fasting Increases Gastrocnemius Muscle and Prefrontal Cortex Thyroid Hormone Levels through Differential BHB and BCAA-Mediated BDNF-mTOR Signaling in Rats
Source: Nutrients. 2022 Mar 10;14(6):1166. doi: 10.3390/nu14061166 (PMC8952016; doi:10.3390/nu14061166)
Supplement: Supplementary file 1 [file nutrients-14-01166-s001.zip › nutrients-1615489-supplementary.pdf]

nutrients MDPI

**Table S1.** ANOVA P-values for each measurement carried out in this study

| FIGURE | Gene-Protein-Compound | ANOVA P value |
|---|---|---|
| **Figure 1 A** | | |
| | BHB | <0.0001 |
| | Leu | <0.0001 |
| | Ile | <0.0001 |
| | Val | <0.0001 |
| **Figure 1 B** | | |
| | mBDNF/ACTIN | 0.0092 |
| | P-TRKB/TRKB | 0.0031 |
| | P-CREB/CREB | 0.0210 |
| | P-AKT/AKT | 0.0094 |
| | P-4EBP1/4EBP1 | 0.0104 |
| | P-P70S6K/ACTIN | 0.0401 |
| | P-FOXO1/FOXO1 | 0.0459 |
| | CASPASE3/ACTIN | 0.0274 |
| **Figure 2 A** | | |
| | T3 (serum) | 0.0015 |
| | T4 (serum) | <0.0001 |
| | T3 (Gastrocnemius muscle) | <0.0001 |
| | T4 (Gastrocnemius muscle) | 0.1824 |
| **Figure 2 B** | | |
| | DIO2 | 0.0026 |
| | DIO3 | <0.0001 |
| | MCT2 | 0.0246 |
| | LAT1 | 0.0055 |
| | LAT2 | <0.0001 |
| **Figure 3 B** | | |
| | BDNF rel. mRNAlevels | <0.0001 |
| **Figure 3 C** | | |
| | mBDNF/ACTIN | 0.0008 |
| **Figure 4 A** | | |
| | BHB | 0.3538 |
| | Leu | <0.0001 |
| | Ile | 0.2078 |
| | Val | 0.0550 |
| **Figure 4 B** | | |
| | P-CREB/CREB | 0.0281 |
| | mBDNF/ACTIN | 0.9829 |
| | P-TRKB/TRKB | 0.0121 |

| | | |
|---|---|---|
| | P-TRKAB/TRKB | 0.0016 |
| | P-AKT/AKT | 0.0012 |
| | P-4EBP1/4EBP1 | 0.0111 |
| | P-P70S6K/ P70S6K | 0.0140 |
| | P-FOXO1/FOXO1 | 0.5699 |
| **Figure 5 A** | | |
| | T3 (Prefrontal cortex) | <0.0001 |
| **Figure 5 B** | | |
| | DIO2 | 0.4829 |
| | DIO3 | <0.0001 |
| | MCT2 | 0.2300 |
| | LAT1 | 0.0102 |
| | LAT2 | 0.6216 |
| **Figure 6 A** | | |
| | MCT8 | 0.9866 |
| | MCT10 | 0.0003 |
| | OATP1C | 0.2352 |
| | OXCT1 | 0.0012 |
| | BDH1 | 0.1050 |
| | BDNF | 0.3550 |
| | TRKB | 0.2187 |
| | PGC1-α | 0.9661 |
| **Figure 6 B** | | |
| | KLF9 | 0.0361 |
| | CALB1 | 0.0340 |
| | HR | 0.0044 |
| | RASD2 | 0.0324 |
| | SEMA3C | 0.0079 |
| | SHH | 0.0266 |
